# PEComa—A Rare Liver Tumor

**DOI:** 10.3390/jcm10081756

**Published:** 2021-04-18

**Authors:** Marek Krawczyk, Bogna Ziarkiewicz-Wróblewska, Tadeusz Wróblewski, Joanna Podgórska, Jakub Grzybowski, Beata Gierej, Piotr Krawczyk, Paweł Nyckowski, Oskar Kornasiewicz, Waldemar Patkowski, Piotr Remiszewski, Krzysztof Zając, Michał Grąt

**Affiliations:** 1 Department of General, Transplant and Liver Surgery, Medical University Warsaw, 02-097 Warsaw, Poland; marek.krawczyk@wum.edu.pl (M.K.); tadeusz.wroblewski@wum.edu.pl (T.W.); piotrek.m.krawczyk@gmail.com (P.K.); oskar.kornasiewicz@wum.edu.pl (O.K.); waldemar.patkowski@wum.edu.pl (W.P.); remi@mp.pl (P.R.); krzysztofzajac85@gmail.com (K.Z.); 2 Department of Pathology, Medical University of Warsaw, 02-097 Warsaw, Poland; bogna.ziarkiewicz-wroblewska@wum.edu.pl (B.Z.-W.); jakub.grzybowski@wum.edu.pl (J.G.); beata.gierej@wum.edu.pl (B.G.); 3 2nd Department of Clinical Radiology, Medical University of Warsaw, 02-097 Warsaw, Poland; joanna.podgorska@wum.edu.pl; 4 Department of General, Gastroenterological and Oncological Surgery, Medical University Warsaw, 02-097 Warsaw, Poland; pawel.nyckowski@wum.edu.pl

**Keywords:** PEComa, rare liver tumor, management, liver resection, perivascular epithelioid cell tumor, surgery, histopathology, mesenchymal tumor, angiomyolipoma

## Abstract

PEComa (perivascular epithelioid cell tumor) is a rare liver tumor. Decisions regarding patient management are currently based on a few small case series. The aim of this study was to report the clinicopathological features of PEComa in order to provide guidance for management, complemented by our own experience. This retrospective observational study included all patients with PEComa who underwent surgical treatment in two departments between 2002 and 2020. A total of 20 patients were diagnosed with PEComa following histopathological examination. The age of the patients ranged from 21 to 73 years. The majority of patients were women (85%). In most patients, the tumors were incidental. In diagnostic studies, PEComas with high arterial vascularization have been described. Liver resection was the treatment of choice. There was only one postoperative complication. During histopathological evaluation, tumors were composed mostly of epithelioid cells, rarely with spindle cell components, thick-walled vessels, and adipocytes in different proportions. Melanocytic markers (HMB45, MelanA) and at least one smooth muscle marker were expressed in all tumors. Features suggestive of malignancy were found in three cases. In conclusion, PEComa is a rare liver tumor that is usually diagnosed incidentally. In radiological studies, tumors with high arterial vascularization are observed. Liver resection is the treatment of choice.

## 1. Introduction

In 1992, Bonetti et al. described a mesenchymal liver tumor derived from perivascular cells for the first time [1]. The name PEComa (perivascular epithelioid cell tumor) was introduced four years later by Zamboni [2]. PEComas are mesenchymal tumors composed of characteristic perivascular cells that show focal association with blood vessel walls and usually express melanocytic and smooth muscle markers [3]. Until recently, PEComa tumors included angiomyolipoma (AML) (PEComa subtype that also contains adipose tissue and thick-walled blood vessels), clear cell/sugar tumor of the lung, lymphangioleiomyomatosis, and other histologically and immunohistochemically similar tumors (e.g., clear cell myomelanocytic tumor) that develop in different locations in the soft tissues and visceral sites [3]. According to the newest edition (the 5th edition) of the WHO 2020 classification, the terms clear cell myomelanocytic tumor and sugar tumor of the lung are not recommended, and epithelioid AML is a synonym for PEComa [4].

PEComa most often occurs in the uterus. It also develops in a wide variety of organs, such as the kidneys, bladder, prostate, lungs, pancreas, and liver [5]. Compared to other liver tumors, these lesions are rare and difficult to recognize. Clinical presentation is non-specific; therefore, these tumors are most often detected accidentally [6,7,8]. Laboratory tests in patients with PEComa did not show any specific abnormalities [9]. Symptoms such as abdominal pain, increased tension of the abdominal wall, constipation, or signs of ileus usually occur in cases involving very large PEComa lesions [10].

PEComa is more common in young women, with a 6:1 female to male ratio [6,11]. Usually, these are single lesions; however, multifocality was also reported more frequently in cases involving malignant lesions [12]. Differential diagnoses include other liver tumors, such as hepatocellular carcinoma (HCC), hemangiomas, focal nodular hyperplasia, and liver adenomas [8,13]. In liver ultrasound examination, computed tomography (CT), and magnetic resonance imaging (MRI), the tumors are often misdiagnosed as HCC. Diagnosis of PEComa should be considered when the tumor looks similar to HCC, the alpha-fetoprotein (AFP) concentration is within normal limits, and there is no history of viral hepatitis or cirrhosis [6,14].

Until now, PEComa tumors reported in the literature were mostly benign; however, malignant tumors have also been reported in a small number of patients. In cases involving malignant lesions, recurrences or metastases were observed in patients with tumors exceeding 5–7 cm in size, with marked nuclear atypia, pleomorphism, high mitotic index, presence of necrosis, and infiltrative margins [4,5]. Malignant PEComa originating from the liver can affect many abdominal organs simultaneously, including the omentum, resulting in massive bleeding into the peritoneal cavity [15].

**Figure 1:**
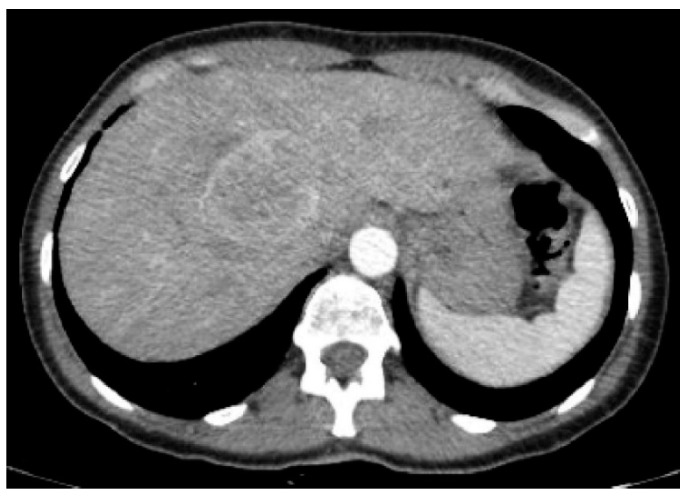
Computed tomography shows tumor with perivascular epithelioid cell tumor with strong contrast enhancement in the arterial phase.

**Figure 2:**
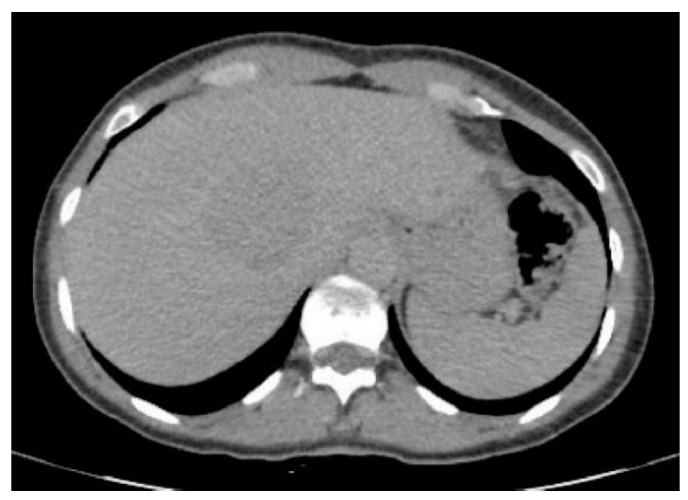
Computed tomography of the patient from Figure 1—“washing out” the contrast—the lesion appears in the delayed phase less attenuated compared to the liver parenchyma.

**Figure 3:**
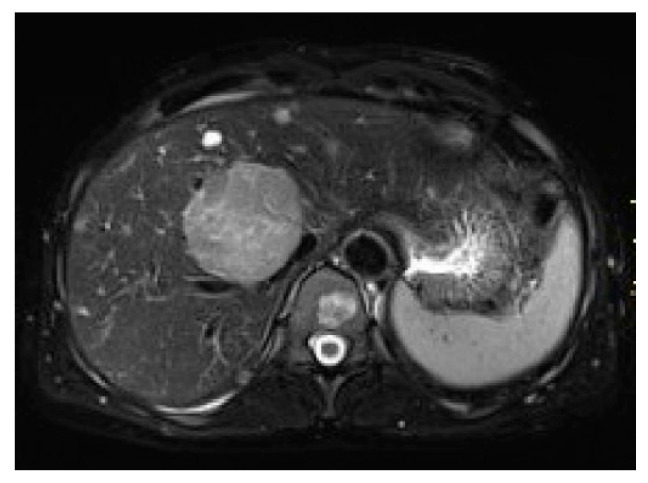
Magnetic resonance, T2 fat saturated images—hyperintensive and heterogeneous lesion.

**Figure 4:**
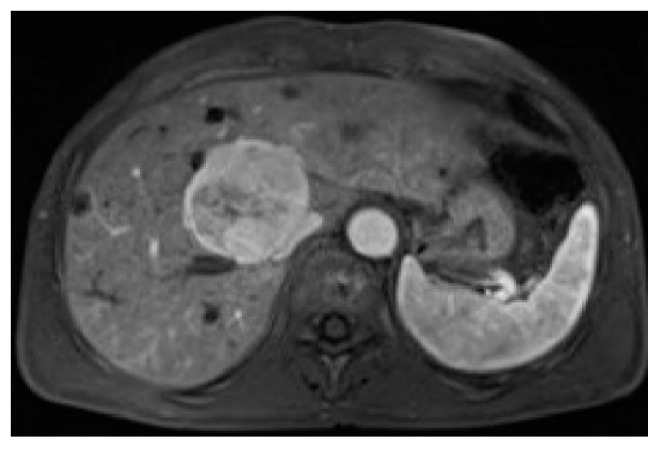
Magnetic resonance, arterial phase images after contrast administration—strong heterogeneous contrast enhancement.

PEComas are tumors with profuse arterial vascularization both inside the tumor and on the periphery, which is why imaging studies using intravenous contrast are more accurate for the evaluation of this type of lesion. It is emphasized that in ultrasound studies, the echogenicity of the tumor can be mixed; however, these tumors are most often hyperechogenic [6,15,16]. In power Doppler imaging, strong vascularization of the lesion can be observed, while in ultrasonography involving intravenous contrast administration, the lesion is strongly and heterogeneously enhanced, followed by rapid contrast drainage into the venous vessels. Similarly, both in CT and MRI, the main features of these tumors are the strong enhancement in the arterial phase and contrast wash-out in the veno-portal and delayed phases, as a consequence of rich vascularization from the branches of the hepatic artery (Figure 1, Figure 2, Figure 3 and Figure 4). The presence of adipose tissue, which is easier to detect on MRI, is typical only for some PEComas, such as AMLs [12]. In addition, the tumor does not show MR-specific features; it is usually hyperintense in T2-weighted images and hypointense in T1-weighted images and may show restriction of water diffusion [6,10,15]. Because the entire radiological image, including possible fat content, is similar to that of HCC or hepatic adenoma, clinical and laboratory information is crucial for differential diagnosis. In the literature, PEComas which did not show contrast “wash-out” have also been noted, which overlap with features of benign, well vascularized tumors such as focal nodular hyperplasias (FNH) and hemangiomas [6]. These, however, should not be mistaken, as FNHs show very homogenous enhancement and in turn, hemangiomas have a typical blood pooling appearance, which both differ significantly from heterogenous enhancement of PEComas. Some overlapping may also be noted with another rare entity: hepatic epithelioid hemangioendothelioma (HEHE), however these, unlike PEComas, are frequently multiple and subcapsular, with capsular retraction and vessels terminating at the edge of the lesions (the lollypop sign).

The final diagnosis is mostly made after histological and immunohistochemical analysis of the resected tumor using the following markers: human melanoma Black-45 (HMB-45), melan-A, MITF, smooth muscle antigen, desmin, and caldesmon [4,17]. Preoperative diagnosis, even in core needle biopsy, is difficult [16].

The basic treatment for patients with PEComa is surgery (excision of the lesion), which in the case of benign tumors (and PEComa, which occurs in the majority of the patients) is sufficient. In malignant lesions, apart from surgical treatment, there is no established post-operative therapy, chemotherapy, or radiotherapy [12,17].

## 2. Materials and Methods

This was a retrospective observational study performed in two surgical departments at the Medical University of Warsaw: the Department of General, Transplant, and Liver Surgery and the Department of General, Gastroenterological, and Oncological Surgery. Consecutive patients with histologically confirmed PEComa who underwent surgical treatment between 2002 and 2020 were included. As this was a retrospective analysis, neither informed patient consent nor ethical committee approval was required according to the national legislation.

Patient baseline characteristics, results of imaging studies, and data on operative management and postoperative courses were obtained from patient medical documentation and internal electronic databases. Survival data were obtained from a national database. Postoperative complications were defined as primary outcome measures. Patient survival was the secondary outcome measure. It was defined as the time between operative management and patient death. Observations were censored at the time of the last follow-up visit.

Histopathological specimens were re-evaluated for the purposes of this study. Data on immunohistochemical analysis results were captured.

Survival outcomes were assessed using the Kaplan–Meier estimator.

## 3. Results

A total of 20 patients were diagnosed with PEComa following histopathological examination in the Department of General, Transplant, and Liver Surgery and the Department of General, Gastroenterological, and Oncological Surgery at the Independent Public Central Clinical Hospital of the Medical University of Warsaw between 2002 and 2020. The age of the patients ranged from 21 to 73 years. The vast majority were women (*n* = 17, 85%). In most patients, the tumor was detected accidentally during ultrasound tests performed for other reasons. One patient with an 8 cm diameter lesion complained of distressing pain in the right costal arch.

**Figure 5:**
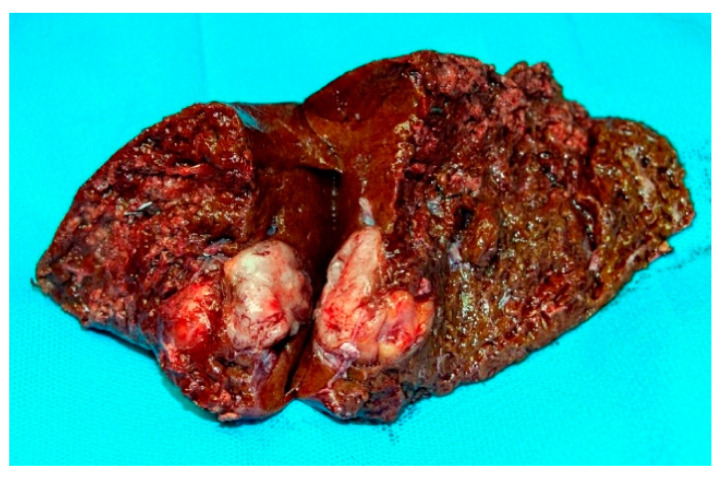
Segments 6 and 7 together with PEComa.

None of the patients had a history of parenchymal liver disease. Laboratory test results were normal. Serum AFP, carcinoembryonic antigen, and Ca 19-9 concentrations were also within the normal limits. Liver resection was the treatment of choice. The scope of resection included anatomical and non-anatomical excisions, and the extent depended on the size and location of the lesions in the liver (Figure 5).

**Figure 6:**
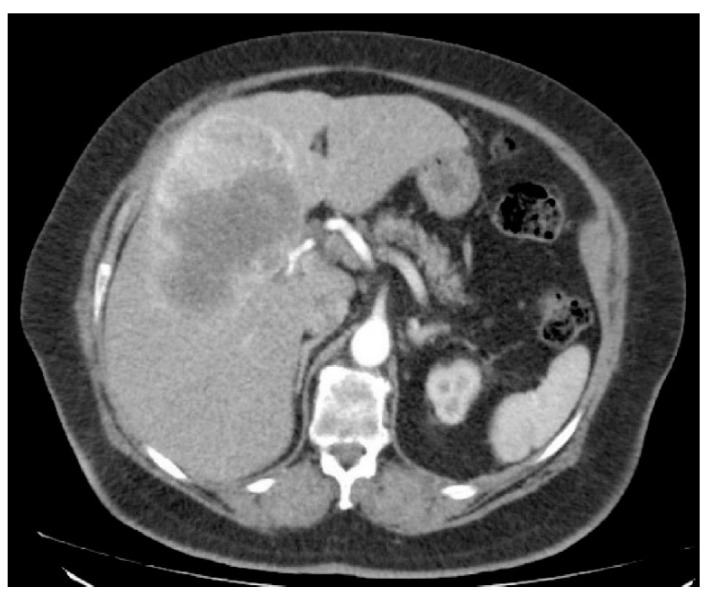
CT of a patient with PEComa. The arterial phase is associated with a strong, heterogeneous enhancement of contrast changes.

**Figure 7:**
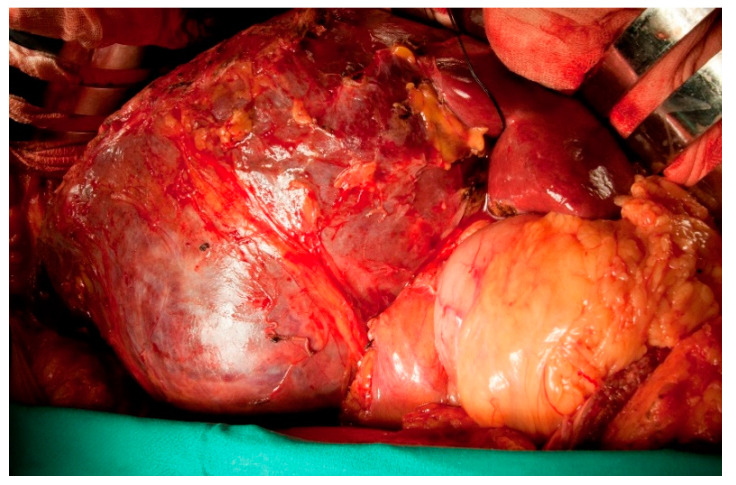
Intraoperative image of PEComa. Tumor segments 5, 6, and 4b reached the round ligament (the same patient as in Figure 6).

**Figure 8:**
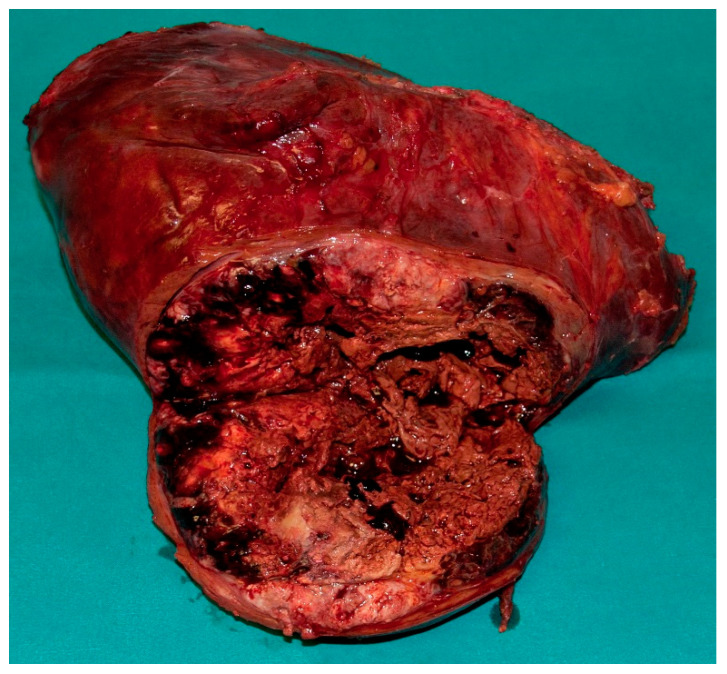
Specimen after resection—the same patient from Figure 6 and Figure 7.

In one patient with an extensive size change of 8 cm (Figure 6), partial hepatic ischemia was performed with resection of segments 4b, 5, and 6 (Figure 7 and Figure 8).

Postoperative complications occurred in one patient who had an external biliary fistula. The patient was treated conservatively.

**Figure 9:**
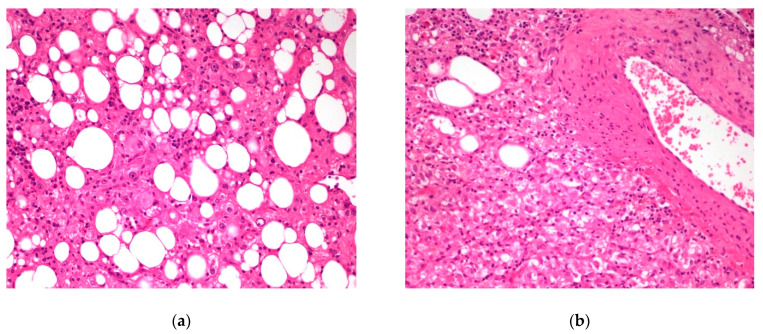
Two images (**a**,**b**) of angiomyolipoma. Hematoxylin & eosin. Objective magnification 20×.

In microscopic studies, 13 tumors (65%) had features consistent with typical AML (Figure 9). They were composed of mature adipose tissue, thick-walled blood vessels, and epithelioid cells with abundant clear or eosinophilic cytoplasm forming nests and trabeculae. The proportions of components varied significantly. In two cases, the adipose tissue was very scarce. The remaining seven tumors did not contain adipose tissue. The size of the tumors was similar in both groups (AML/PEComa lacking adipocytes). No differences were found in mitotic activity, cytological atypia, and necrosis between the two groups.

Extramedullary hematopoiesis was observed in 7/20 tumors. The spindle cell component was found in four cases; however, it was never dominant and was not related to the other morphological features of the tumors.

**Figure 10:**
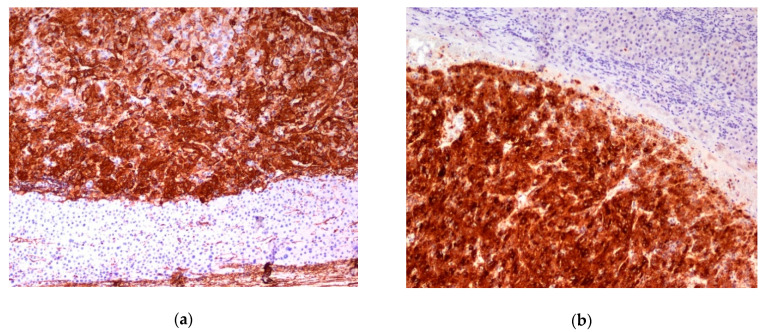
Two examples (**a**,**b**) of positive immunohistochemical stain with smooth muscle antigen and Melan A. Objective magnification 10×.

Immunohistochemical features were typical. Melanocytic markers (HMB45, MelanA) and at least one smooth muscle marker were expressed (Figure 10).

**Figure 11:**
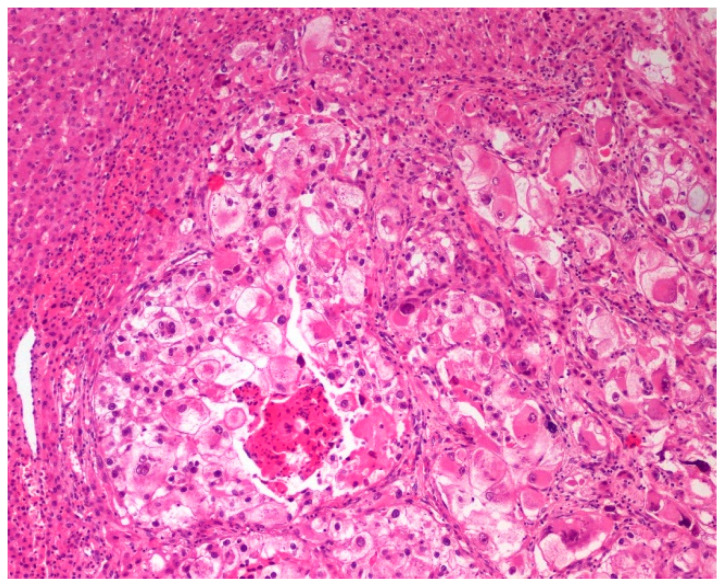
PEComa—infiltrative growth of the tumor. Objective magnification 10×.

In three cases, at least three of the following features potentially suggesting malignancy were found: size ≥ 5 cm, high-grade atypia, mitoses > 1/50 high-power fields, necrosis, infiltrative growth, and lymphovascular invasion (Figure 11) [18].

**Figure 12:**
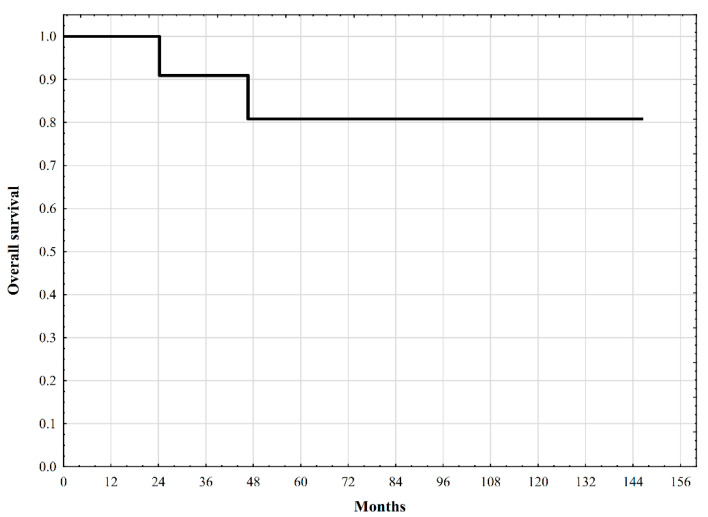
Overall survival of patients with PEComa undergoing surgical treatment.

Two patients died during the follow-up period, with one-, three-, five-, and ten-year survival estimates of 100%, 90.9%, 80.8%, and 80.8%, respectively. The survival curve is shown in Figure 12.

## 4. Discussion

In the literature, PEComa tumors of the liver have been reported for 28 years. Most reports are based on individual cases [2,7,8,11,15,17,19]. Only two publications presented a larger series: seven cases in the first publication [5] and 13 cases in the second publication [14]. Our study included 20 patients who underwent surgery in two surgical departments of the Medical University of Warsaw, making it one of the studies with the largest sample size from one institution.

Some of the issues that were reported in the published studies were confirmed in our study. Patients admitted to both departments had no established diagnosis. In most cases, the lesions resembled HCC on CT and MRI [8,13]. All patients treated in our center developed hepatic lesions even though they had healthy livers, did not have a history of hepatitis, and did not have any factors that damage the parenchyma. These characteristics were also observed by other authors [6,14]. Similar to other published studies, the patients in our study were mostly female patients, constituting 85% of the group.

CT of the arterial phase showed intensive saturation of the vessels in the periphery of the tumor, with a marked decrease in the amount of contrast in the portal and delayed phases. In MR studies in the T1 phase, a significant intensity was visible, while the T2 intensity decreased. Similar observations have been reported in the literature [6,10,15].

Patients were qualified for surgical treatment based on the results of their radiological examination, which, although did not provide a clear diagnosis, indicated the need for surgical treatment. The extent of liver resection was dependent on the location and size of the tumor. In our opinion, there is no need for extensive liver resection or hemihepatectomy. Most patients underwent non-anatomical resection. However, we have always strived for oncological completeness. The oncological margin in the patient with the largest tumor (8 cm) was only 1 mm. This resection, with such a small margin, was performed due to the size of the tumor, and the non-expanded segments of the tumor that did not contain cancerous cells, which remained after the resection should be sufficiently large in relation to patient’s body weight.

None of the patients in the reported series underwent preoperative biopsy. The decision on surgery was based on the results of the imaging studies. We assumed that the patients had indications for resection and a biopsy would not change our decision. In other series, a biopsy was however performed. For example, out of seven treated patients, the authors [9] performed a fine-needle biopsy in two, finding cells suggestive of PEComa, but the result did not change their decision and they performed a liver resection. While non-anatomical resection is sufficient for patients with PEComa, anatomical resection may be superior over non-anatomical resection in the case of HCC diagnosis [20,21,22,23]. Nevertheless, patients with PEComa typically have no underlying liver disease, which makes this population more similar to patients with HCC occurring in a healthy liver. In the latter, achieving an R0 resection status seems to be of the most importance [24].

Histopathological diagnosis of PEComa is based on morphological and immunohistochemical features that allow differentiation of the tumor from other focal lesions in the liver, including HCC. Pecoma, as a neoplasm with myomelanocytic differentiation, typically expresses both melanocytic (HMB-45, MelanA, Mitf) and myogenic markers (actin, myosin, calponin) with variable intensity and distribution of staining [4]. TFE3 is a new marker that is strongly expressed in 15% of PEComas, indicating the presence of TFE gene rearrangement. Pecomas containing TFE3 gene rearrangements stain strong with HMB45 and TFE 3, but the expression of MelanA and smooth muscle markers tend to be focal or negative [18]. Patients with this abnormality are younger. Microscopically, the tumors have an alveolar pattern, consist of epithelioid cells, and often do not express smooth muscle markers [4].

Molecular genetic data on PEComas are limited, but nowadays, two distinct molecular groups have been described: PEComas with TSC2 mutations and tumors with TFE3 fusions (TFE3-translocation associated PEComas). Furthermore, TP53 mutations have been identified in 63% of the TSC2 mutated Pecomas. The patients of the first group (if diagnosed with malignant tumors) may benefit from targeted therapy with mTOR inhibitors [4,25]. RAD51B fusions and HTR4-ST3GAL1 fusion are other rearrangements found in PEComas [18,26].

One study described a lesion that was different from primary lesions in the liver. The aforementioned study involved a young man who underwent surgery for a rectal tumor, which turned out to be a PEComa following histopathological analysis. The patient was treated surgically, and anterior low rectal resection was performed. Four years later, a small metastatic lesion was diagnosed in the liver. The patient agreed to the surgery four years later, after the number of lesions had increased to three and the size had increased. Non-anatomic liver resection with confirmed PEComa metastasis was performed, followed by a satisfactory postoperative course [19].

Surgical treatment of patients with primary liver PEComa is the treatment of choice and is effective. The number of postoperative complications was relatively small. Nevertheless, these patients require constant oncological supervision similar to patients with cancerous tumors in the liver.

In the case of disseminated malignant PEComas, chemotherapy becomes the treatment of choice, considering the potential effectiveness of mTOR inhibitors [27,28]. There are very few reports on non-surgical treatment of patients with liver PEComas. In rare situations, transarterial chemoembolization and ablation have been applied [9,29]. Those non-surgical methods should be considered in case of contraindications for surgery.

## 5. Conclusions

PEComa is a rare liver tumor that is usually diagnosed incidentally. In radiological studies, as a tumor with high arterial vascularization, it is necessary to differentiate it primarily from HCC. The treatment of choice is surgery, that is liver resection. The final diagnosis is confirmed only after histopathological examination of the resected specimen and requires a number of immunohistochemical tests.

## Data Availability

The data is available from the Authors upon reasonable request.
